# Factors associated with family function in school children: Case-control study

**DOI:** 10.1016/j.heliyon.2023.e14595

**Published:** 2023-03-16

**Authors:** Alejandro Botero-Carvajal, Ángela María Jiménez Urrego, Ana G. Gutierrez-Posso, Mario Calero-Flórez, Mauricio Hernandez-Carrillo

**Affiliations:** a Universidad Santiago de Cali, Cali, Colombia; b Universidad de San Buenaventura seccional Cali, Cali, Colombia; c Master in Mental Health of Children and Adolescents, Professor at the Pontificia Universidad Javeriana Cali, Colombia; d Fundación Universitaria San Martin Cali, Cali; Colombia; e Universidad del Valle, Universidad Libre, Escuela Nacional del Deporte, Cali, Colombia

**Keywords:** Family relations, Risk factors, Control groups

## Abstract

**Background:**

Family functionality is that which promotes the integral development of its members, as well as a favorable state of health in them; fulfilling the basic parameters of adaptation, participation, growth, affection, and resources. Family dysfunction is understood as the failure to comply with any of these functions due to an alteration in one or more of the subsystems.

**Objective:**

There is little research on variables related to family functioning with a case-control design, for this reason, we present the family functioning of school students, identify variables found to be related to family functioning, and describe a model of variables related to family dysfunction.

**Materials and methods:**

Analytical study of cases and controls. The sample was made up of 290 students. The APGAR scale was used to identify family dysfunction. The statistical processing was done in Epi-Info 7.0 and STATA 14.

**The variables that were considered were:**

Municipality, area, age, sex, school grade, mother's age, disability, and displacement.

**Results:**

The factors associated with adequate family function were: displacement, (OR = 0.17, CI: 0.03–0.99). You are followed, your parents pay attention and listen to you (OR = 0.25, CI: 0.08–0.74), you talk to your mother every day (OR = 0.35 CI: 0.16–0.74), you spend free time with your parents (OR = 0.41, CI: 0.20–0.86), play sports at least once a week (OR = 0.42, CI = 0.20–0.91), and finally, attend religious services (OR = 0.51, CI: 0.29–0.90). While the factors associated with family dysfunction were: your parents punish you by forbidding you things (OR = 2.98, CI: 1.32–6.71) and you have friends close to where you live followed by an (OR = 2.60, CI: 1.13–5.96).

**Conclusions:**

Dysfunctionality was evident in the four municipalities of the Valley. Among the main factors associated with dysfunctionality was punishment by parents for forbidding things and having friends near the schoolchild's home.

## 1 Introduction

The emphasis on the family can be traced back to social system theoretical models [1], in the growing interest in understanding how family members exhibit behaviors and interactions with each other [2]. Interactions are understood within a system, in which the family adjusts to the subsystems in which it interacts, adapting to the social rules of the time [3].

The family theory is assumed as a system, in which the interaction between family members defines patterns of health and illness among members. For this reason, identifying which factors affect family functioning is key because family functioning reflects the social structure of the family environment, related to levels of cohesion, support, conflict, organization, and quality of communication. A functional family is one where the family environment allows for clear communication, affective regulation, defined roles, and cohesion. On the contrary, family dysfunction is a family environment characterized by the absence of defined roles, a high level of conflict, and poor regulation of behavior and emotions [4,5].

## 2 Family functioning and APGAR

This set of elements related to decision making and family support among family members is known as family functioning. Family functioning is established according to the degree of compliance with the basic parameters of family functioning (APGAR): Adaptation, Participation, Gain or Growth, Affection, and Resources [6, 17].

The instrument investigates the perception of each component of the family function on a Likert-type scale. The adaptation describes the use of internal and external family resources to solve problems affecting family balance. Participation refers to the sharing of responsibilities and how shared decisions are made. Likewise, gain or growth is the reciprocal support that helps family members to self-actualization, and emotional and physical maturity. On the other hand, affection expresses the relationships of love and care within the family. Finally, resources describe the decisions to share physical space, health, time, and other resources with family members [7].

From an evaluative perspective of the family function [4], how family members perceive their functioning is addressed in recent studies with the family APGAR, due to its psychometric properties and sensitivity in the screening of family members [[8], [9], [10], [11]].

Family functioning is related to health status [12] where the family influences the health process of its members, according to the decisions made within the family group about the beliefs transmitted and the type of help provided [13, 14, 25, 56].

## 3 Family dysfunction has been linked to mental health issues

Previous studies point to the relationship between family dysfunction and mental health [[15], [16], [17]]. Subjects with a history of family dysfunction are more vulnerable to sexual abuse and neglect [18,19]; relationships between family dysfunction and emotional intelligence [20]; academic achievement [21]; effectiveness of family functionality [22]; usefulness of family APGAR in identifying family function [7,23].

However, although the relationship between family function and health has been widely documented, there is insufficient evidence as to what factors are associated with its functioning. For this reason, we present the factors associated with family function and family dysfunction in four municipalities of Valle del Cauca.

In conclusion, spending free time with the parents, talking every day with the mother, attention and listening on the part of the parents, attending a religious service, and playing sports at least once a week are factors associated with family functioning. While prohibitive punishments by parents, and having friends close to where you live, are factors associated with family dysfunction.

## 4 Materials and methods

This was an analytical case-control study. A secondary source used in a previous study was used [24].

## 5 Participants

The study population came from the secondary database provided by the Departmental Health Secretariat, which is made up of school children enrolled in grades 6 to 11, who attended official educational institutions in the municipalities of Candelaria, Florida, Jamundí, and Pradera. Valle del Cauca, Colombia.

## 6 Instruments

In the case study, a questionnaire was applied that contained questions about the family relationship and the social environment of the schoolchild. The APGAR scale was used to identify family dysfunctions. Cronbach's alpha for the family APGAR was 0.90 in Colombia, suggesting that it represents unidimensional construct validity: family function [25].

The items of the scale are scored as never, rarely, sometimes, almost always, and always. Total scores range from 0 to 20; the higher the score, the better the family functionality [26].

The family relationship was measured using dichotomous questions, about living with their parents, free time shared with them, and talking with them daily; in addition, the physical and verbal punishment that was administered in the upbringing of the schoolchild was investigated.

The variables that were taken into account were: Municipality, area, age, sex, school grade, mother's age, disability, and displacement. The social environment was measured using questions about their immediate environment in terms of place of residence.

There was no bias in the identification of the exposure since the items measured were provided in the same way and confidentiality was guaranteed at the time of completion. As for the measurement of the result (family dysfunction), there was the disadvantage of lack of sample to determine a cut-off point to avoid bias of bad classification, given the continuity of the score in the identification of bullied schoolchild and non-bullied schoolchild according to the CISNEROS self-test GI.

### 6.1 Procedure

The family APGAR is a test carried out in the form of a questionnaire and consists of 5 questions. The participant must select the degree to which he or she perceives to be within his or her family unit. The score ranges, for each of the 5 items, between 0 and 4 points, following a classification of five options that varies from "never" to "always", so that the minimum possible score would be 0 and the maximum 20:

Normal family function: 17–20 points.

Mild dysfunction: 16–13 points.

Moderate dysfunction: 12–10 points.

Severe dysfunction: 9 points or less.

Considering the evaluation of the APGAR Family, we defined as cases (students with family dysfunction) those school children who presented scores less than or equal to 16, and controls (students without family dysfunction) with scores greater than 16 points. The distribution of the sample is presented in Table 1.

**Table 1:** Distribution of the sample, by the municipality of the school children enrolled in grades 6 to 11 in four municipalities in Valle del Cauca, Colombia. The year 2009.

Municipality	Total students registered in the database	Cases	Controls	Total
Candelaria	307	40	40	80
Florida	270	42	42	84
Jamundí	344	48	48	96
Pradera	220	15	15	30
Total	1141	145	145	290

A 1:1 analysis was conducted, paired by the municipality. This study was carried out from an analytical approach of paired cases and controls, the raw and adjusted Odds Ratio (OR) were calculated (using conditioned logistic regression).

A descriptive exploratory analysis was carried out to evaluate the behavior of the variables and to identify possible extreme values, which were not presented in the present analysis; therefore, the behavior of these variables was within the expected values according to the literature. To evaluate the different exposures from the sociodemographic and social fields, a bivariate analysis was carried out using statistical tests (with probability values lower than 0.05) and by calculating raw OR to describe the relationship of the dependent variable (IE) with the exposure variables.

To determine which variables were included in the logistic conditioned regression model, a statistical criterion was taken into account, and the variables that presented statistical significance were included with p ≤ 0.2. The 0.2 criteria are used for the input of variables in the modeling process, following the procedure described in previous studies (Aguayo-Albasini et al., 2014; [24].

The aim was to obtain a parsimonious model that had an adequate classification of family dysfunction and that also presented statistical significance in the epidemiological association of its variables.

Several models were made with the variables that presented statistical significance in the bivariate analysis of family dysfunctionality in school children. Each new model was compared with the immediately previous one using the log-likelihood test, the Pseudo R2, the Akaike's Information Criterion (AIC), and the Bayesian Information Criterion (BIC), to determine the model's fit according to the variables included. The most parsimonious model was selected, with the best fit and with the epidemiological association in most of its explanatory variables.

Opportunity reasons (OR) with IC95% were determined, to establish the association between the variables contained in the database and the presence of family dysfunction. The data were processed in the statistical package R version 3.5.0.

### 6.2 Ethical considerations

We had the authorization of the Departmental Public Health Secretariat of Valle del Cauca for the use of the database. The research was considered safe according to resolution No. 008430 of 1993. And informed consent was obtained from all participants. At no time was the identity of the participants known, nor was any information available that would allow the schoolchild who filled out the questionnaire to be recognized. The research was approved by the Ethics Committee of the Universidad San Martín, Cali.

## 7 Results

The participants in the study were students assigned to the Departmental Health Secretariat; they were 1141 students enrolled in grades 6 to 11, between the ages of 10 and 18, attending official educational institutions in the municipalities of Candelaria, Florida, Jamundí, and Pradera, in Valle del Cauca, a region located in the southwest of Colombia. A questionnaire containing questions on sociodemographic information, family relationship, and the social environment of the schoolchild was applied.

In the bivariate analysis, the variables were compared according to cases and controls, and no statistically significant differences were found in the variables: sex, grade, stratum, disability, displacement, and age in both sexes. While for the variable's area of residence and bullying differences were obtained, which indicates that for these two variables there was different participation in these groups for these two variables (Table 2).

**Table 2:** Bivariate analysis. School children enrolled in grades 6 to 11 in four municipalities in Valle del Cauca, Colombia. The year 2009.

Variable/category		Cases		Controls		P value
		n = 57		n = 233		
		n (%)		n (%)		
Gender	F	34 (59,6)		116 (49,8)		0,182
	M	23 (40,4)		117 (50,2)		
Grade	10° y 11	14 (24,6)		36 (15,5)		0,250
	6° y 7°	23 (40,4)		111 (47,6)		
	8° y 9°	20 (35,1)		86 (36,9)		
Stratum	Low	52 (91,2)		204 (87,6)		0,440
	Medium	5 (8,8)		29 (12,4)		
Zone	Rural	27 (47,4)		147 (63,1)		**0,030**^a^
	Urban	30 (52,6)		86 (36,9)		
Disability	No	50 (87,7)		217 (93,1)		0,175
	Yes	7 (12,3)		16 (6,9)		
Displaced	No	56 (98,2)		225 (96,6)		0,512
	Yes	1 (1,8)		8 (3,4)		
Bullying	No bullying	20 (35,1)		125 (53,6)		**0,012**^a^
	Yes bullying	37 (64,9)		108 (46,4)		
Variable/category		n	average (SD)	n	average (SD)	P value
Age	Female	34	13,61 (1,93)	116	13,48 (1,86)	0,7228
	Male	23	13,30 (1,91)	117	13,07 (1,46)	0,5139

a p-value <0.05.

**Fig. 1:**
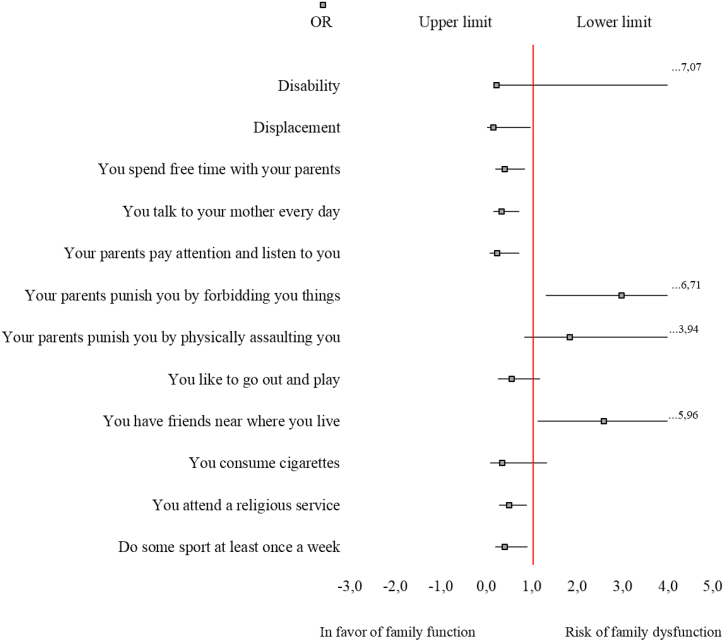
Risk model for family dysfunction.

The final model was constituted by 12 independent variables of which 8 presented statistical significance, 6 were related to a good family function and 2 to family dysfunction, and the other 4 presented no association (Fig. 1).

The factors associated with good family function were: displacement, (OR = 0.17, CI: 0.03–0.99). You are followed, your parents pay attention and listen to you (OR = 0.25, CI: 0.08–0.74), you talk to your mother every day (OR = 0.35 CI: 0.16–0.74), you spend free time with your parents (OR = 0.41, CI: 0.20–0.86), play sports at least once a week (OR = 0.42, CI = 0.20–0.91), and finally, attend religious services (OR = 0.51, CI: 0.29–0.90). While the factors associated with family dysfunction were: your parents punish you by forbidding you things (OR = 2.98, CI: 1.32–6.71) and you have friends near where you live followed by an (OR = 2.60, CI: 1.13–5.96) (Table 3).

**Table 3:** Multivariate analysis using conditional logistic regression Factors associated with family dysfunction in school children enrolled in grades 6 to 11 in four municipalities in Valle del Cauca, Colombia Year 2009.

Significant variables	OR	p-value	I·C. 95%
Disability	0.23	0.116	0.80–7.07
Displacement	0.17	0.049*	0.03–0.99
You spend free time with your parents	0.41	0.018*	0.20–0.86
You talk to your mother every day	0.35	0.006*	0.16–0.74
Your parents pay attention and listen to you	0.25	0.013*	0.08–0 .74
Your parents punish you by forbidding you things	2.98	0.008*	1.32–6.71
Your parents punish you by physically assaulting you	1.84	0.117	0.85–3.94
You like to go out and play	0.56	0.135	0.26–1.19
You have friends near where you live	2.60	0.024*	1.13–5.96
You consume cigarettes	0.36	0.130	0.09–1.34
You attend a religious service	0.51	0.020*	0.29–0.90
Do some sport at least once a week	0.42	0.029*	0.20–0.91

* p-value <0.05.

## 8 Discussion

Family characteristics are shown to be the most significant in predicting mental health problems [27]. The importance of research lies in pointing out the protective and risk factors for family dysfunction. Among the protective factors for family dysfunction, forced displacement is surprising as a finding, given that it has been linked to adverse effects on populations: increase in delinquency [28]; rupture of social and labor ties, stress, and psychophysical and identity vulnerability [29]. However, the research points to forced displacement as a means of protecting families from family dysfunction, which is consistent with previous research that indicates that, in the face of critical situations associated with displacement, families express support and affection among their members [30].

In addition, the findings of the research indicate the behaviors to be promoted in the family, that is, the dimensions of Adaptation, Participation, Gain, Affection, and Resources, contemplated in the family APGAR can be oriented towards participation in public policies that, in terms of the use of free time, sports practices, and the promotion of good treatment within the family, are available in the municipalities and departments. Given in addition to travel, sharing free time with the school and the parents; talking daily with the mother; attention to the parents and listening to the children; doing some sport at least once a week, and attending a religious service, are protective factors for family dysfunction. In that order of ideas, the protective factors found for family functionality become developmental objectives for national, regional, and local development plans.

It should be noted that sharing free time with parents is a protective factor. Previous research indicates benefits for the psychological development of children when parents share time with them, improving the family climate, increasing emotional ties, and increasing confidence and self-esteem [31], which suggests that schoolchildren who spend free time with their parents increase the spaces for family interaction and favor the practice of each dimension together with the monitoring of the schoolchildren's free time. This finding also implies that parental company, i.e., decreasing the time the schoolchild is alone, protects them against health problems, such as tobacco use [32] and loss of sleep [33].

Talking to the mother every day is an element that favors communication within the family, besides favoring family functioning, she warns that better communication prevents adverse consequences for mental health [34].

This is related to our finding on paying attention and listening on the part of parents, because, as has been insisted so far, the dimensions of family functioning are crossed by the perception that the member has on whether he is listened to, supported, by his family. Thus, recommending paying attention and listening to children favors family functioning, similar to recent studies by Ref. [35].

Likewise, religious service favors family functioning, we believe for two main reasons, the first emphasizing family functioning for the functioning of the individual and the second on the characteristics of the individual, on moral aspects of his or her behavior within the family group. These reasons are reported in some studies that relate religious aspects with parental practice [36,37].

Although the study found that playing sports at least once a week is a factor associated with family functionality, the literature indicates that there is no association with family dysfunction in playing or not playing sports, but it does indicate that the influence of parents is decisive in the practice of sports by their children [38].

However, we think that the finding is related to the use of free timestamp [39], together with the fact that sports practice emulates the relational structure of society, especially in collective sports. Similarly, there is evidence that the practice of sports contributes to the improvement of mental health, in this framework better states of mental health allow a better relationship with others and with oneself [[39], [40], [41], [42]].

Among the factors associated with family dysfunction, punishment through prohibition was shown to be a risk factor, as was having friends near one's place of residence, which coincides with previous studies [6,[43], [44], [45], [46], [47]].

Physical punishment shows a negative relationship with family functioning, explained in its negative results for mental health, which intervenes in the recognition of oneself and others, a necessary aspect in social cognition for the maintenance of healthy interpersonal relationships camp [48,49]; physical punishment is frequent in the rural Colombian population, similar to the population included in the study camp [50].

Although friends near the place of residence in the study are a risk factor associated with family dysfunction, the literature is consistent in pointing out that it is characteristic of dysfunctional families that their children present negative characteristics related to the degree of emotional maturity, mental health disorders, difficulty in building healthy relationships with peers, SPA addiction, conflict with authority, school difficulties, and communication difficulties [51].

We believe that this is related to the time of interaction of the minor with the family, in which during adolescence the child seeks to belong to a reference group, which in most cases is outside the family. This finding is similar to that found in other studies that suggest that communication and family dynamics change throughout the course of life and the development process of the member's [52], as well as the current social identity, which explains new readings on the processes of socialization and belonging to the group, which include the dynamics of social networks, which surpasses the concept of having friends close to the physical place to have friends from anywhere in the world [53].

## 9 Recommendations and limitations of the study

The literature refers that family support is related to mental health [54], therefore, family intervention programs can be oriented towards promoting the variables found in the study as significant for family functionality.

It is significant to note that aspects such as the educational level of the mother, the communication of feelings of anger or sadness, and the degree of behavioral regulation affect the child-rearing practices of women who are victims of forced displacement and that for future research these are important variables to include, given that the higher the educational level of the mothers, the greater the assertiveness in child-rearing practices, the greater the manifestation of feelings of anger or sadness, the less communication between mother and child and the greater the regulation of the children's behavior by mothers who have suffered forced displacement [30].

Social support, a topic not explored in the research, is related to family functionality [55] and should be included in future studies. As well as the modulating role that social networks can have as a mechanism of contemporary social interaction.

## Funding sources

This research has been funded by Dirección General de Investigaciones of Universidad Santiago de Cali under call No. 01-2022.
