# Supplementary material for: Factors associated with family function in school children: Case-control study
Source: Heliyon. 2023 Mar 16;9(3):e14595. doi: 10.1016/j.heliyon.2023.e14595 (PMC10031484; doi:10.1016/j.heliyon.2023.e14595)
Supplement: Multimedia component 1 [file mmc1.docx]

**QUESTIONNAIRE ON SCHOOL COEXISTENCE**

**Interviewer ID No.: ___________________ Survey No.________**

This is a questionnaire that has the objective of knowing what coexistence is like with your classmates within your Educational Institution. It is about counting on your answers to identify difficult situations and be able to contribute to their solution. WE ASK YOU TO ANSWER HONESTLY.

# PLEASE ANSWER ALL THE QUESTIONS. THANK YOU!

Educational institution: _______________________________________________________________________

Full name: _______________________________________________________________________

Age: ____ years Sex: F ( ) M ( ) Date of birth (day/month/year): ___/___/______ School grade: _____

Municipality: _________________________ Commune (only for Cali): _____ Stratum: _____

Zone: urban( ) Rural ( ) Father's age: _____ Mother's age: _____

Father's studies: None ( ) Primary ( ) Secondary ( ) Technicians ( ) University students ( )

Studies of the mother: None ( ) Primary ( ) Secondary ( ) Technicians ( ) University students ( )

Father's occupation: _________________________ Mother's occupation: __________________________

Number of members that make up the family (understanding family, parents and siblings ): _____ people

What race do you belong to: Black ( ) Indigenous ( ) Mestizo-White ( ) Other ( )

Do you have a disability: Yes ( ) No ( ), mention which one _____________________________________________

Taking your peers as a reference, you consider yourself to be: Tall ( ) Medium ( ) Short ( )

Have you or your family been displaced in the last 5 years: Yes ( ) No ( )

In which year did you enter the Educational Institution: 2003( ) 2004( ) 2005( ) 2006( ) 2007( ) 2008( ) 2009( )

**For each question, mark only one X, the one that seems to apply to you.**

| **PLEASE ANSWER HONESTLY** | **YES** | **NO** |
| --- | --- | --- |
| You live with your 2 parents |  |  |
| Do you live with someone other than your parents? |  |  |
| Do you have siblings? |  |  |
| You spend free time with your parents |  |  |
| You talk to your mother every day |  |  |
| You talk to your father every day |  |  |
| Your parents pay attention to you and listen to you |  |  |
| Your parents punish you by forbidding you things |  |  |
| Your parents punish you by physically assaulting you |  |  |
| Your parents know your friends |  |  |
| In your home they are physically assaulted |  |  |
| In your home they are verbally assaulted |  |  |
| Your neighbors physically assault each other |  |  |
| Your neighbors verbally assault each other |  |  |
| Do you like to go outside to play? |  |  |
| Do you have friends near where you live? |  |  |
| Your friends are older than you |  |  |
| Your friends drink alcohol |  |  |
| Your friends consume cigarettes |  |  |
| Do you like violent movies? |  |  |
| you consume alcohol |  |  |
| you smoke cigarette |  |  |
| Do you consume any psychoactive substance? |  |  |
| Do you belong to a gang? |  |  |
| Do you attend a religious cult? |  |  |
| Do you do sports at least once a week? |  |  |
| Are you afraid of going to class? |  |  |

| **INDICATE HOW FREQUENTLY THESE BEHAVIORS ARE OCCURRED BY YOUR PARTNERS TOWARDS YOU, IN THE EDUCATIONAL INSTITUTION** | **Never** | **Rarely** | **Many times** |
| --- | --- | --- | --- |
| They do not speak to me |  |  |  |
| They ignore me, they don't listen to me |  |  |  |
| They make me look ridiculous in front of others |  |  |  |
| they won't let me talk |  |  |  |
| They won't let me play with them |  |  |  |
| they call me by nicknames |  |  |  |
| They threaten me to do things I don't want |  |  |  |
| They force me to do things that are wrong |  |  |  |
| they have me mania |  |  |  |
| They don't let me participate, they exclude me |  |  |  |
| They force me to do things that are dangerous to me |  |  |  |
| They force me to do things that make me sick |  |  |  |
| They force me to give them my things or money |  |  |  |
| They break my stuff on purpose |  |  |  |
| they hide things from me |  |  |  |
| They steal my things |  |  |  |
| They tell others not to be or not to talk to me |  |  |  |
| They forbid others to play with me |  |  |  |
| They insult me |  |  |  |
| They make gestures of mockery or contempt towards me |  |  |  |
| They don't let me talk or interact with others |  |  |  |
| They prevent me from playing with others |  |  |  |
| I get slapped, punched, kicked. |  |  |  |
| they yell at me |  |  |  |
| They accuse me of things I haven't said or done |  |  |  |
| They criticize me for everything I do |  |  |  |
| They laugh at me when I'm wrong |  |  |  |
| They threaten to hit me |  |  |  |
| they hit me with objects |  |  |  |
| They change the meaning of what I say |  |  |  |
| They mess with me to make me cry |  |  |  |
| They lash out at me to make fun of me |  |  |  |
| They mess with me because of my way of being |  |  |  |
| They mess with me because of my way of speaking |  |  |  |
| They pick on me for being different |  |  |  |
| They make fun of my physical appearance |  |  |  |
| They go around telling lies about me |  |  |  |
| They try to make others feel bad |  |  |  |
| they threaten me |  |  |  |
| They wait for me at the exit to mess with me |  |  |  |
| They make gestures to scare me |  |  |  |
| They send me messages to threaten me |  |  |  |
| they push me to intimidate me |  |  |  |
| They behave cruelly with me |  |  |  |
| They try to punish me |  |  |  |
| they despise me |  |  |  |
| They threaten me with weapons |  |  |  |
| They threaten to harm my family |  |  |  |
| They try to hurt me in everything |  |  |  |
| they hate me for no reason |  |  |  |

***SELF-TEST CISNEROS***

| **RIGHT NOW …** | **Never** | **Sometimes** | **Many times** | **Always** |
| --- | --- | --- | --- | --- |
| I feel that I am a dignified person, at least as much as the others |  |  |  |  |
| I am convinced that I have qualities |  |  |  |  |
| I am capable of doing things as well as most people |  |  |  |  |
| I have a positive attitude towards myself |  |  |  |  |
| In general, I am satisfied with myself |  |  |  |  |
| I feel like I don't have much to be proud of |  |  |  |  |
| In general, I am inclined to think that I am a failure |  |  |  |  |
| I wish I could feel more respect for myself |  |  |  |  |
| There are times when I really think I'm useless |  |  |  |  |
| I often think I'm not a good person |  |  |  |  |

***ROSENBERG SCALE***

| CONSIDERING ONLY HOW YOU HAVE FEELING DURING THE PAST SIX MONTHS | **Never** | **Almost**  **Never** | **Some**  **Times** | **Almost**  **Always** | **Always** |
| --- | --- | --- | --- | --- | --- |
| I feel satisfied with the help I receive from my family when I have a problem and/or need |  |  |  |  |  |
| I am satisfied with the way my family talks about things and shares problems with me |  |  |  |  |  |
| I feel satisfied with the way my family accepts and supports my desire to undertake new activities |  |  |  |  |  |
| I feel satisfied with the way my family expresses affection and responds to my emotions such as anger, sadness or love |  |  |  |  |  |
| I feel satisfied with the way we share in my family: time to be together, spaces at home or money |  |  |  |  |  |

***familial apgar***

| **DURING THE LAST TWO WEEKS** | **Never** | **Sometimes** | **Many times** | **Always** |
| --- | --- | --- | --- | --- |
| I have felt happy and in good spirits |  |  |  |  |
| I have felt calm and relaxed |  |  |  |  |
| I feel active and energetic |  |  |  |  |
| I have woken up feeling well and rested |  |  |  |  |
| My daily life has had interesting things for me |  |  |  |  |

***Well-Being Index (WHO Well-Being Index, WHO-5)***

| **DURING THE LAST TWO WEEKS** | **Never** | **Sometimes** | **Always** |
| --- | --- | --- | --- |
| Have you had difficulty sleeping? |  |  |  |
| Have you been easily frightened or alarmed? |  |  |  |
| Have you felt nervous or tense? |  |  |  |
| Have you felt sad? |  |  |  |
| Has it been difficult for you to enjoy your daily activities? |  |  |  |
| Have you felt tired? |  |  |  |
| Have you stopped attending classes? |  |  |  |
| Have you stopped interacting or carrying out activities with your relatives? |  |  |  |
| Have you stopped relating to people in your social group or friends? |  |  |  |
| Have you felt that you have emotional problems and that you require professional attention? |  |  |  |

***Personal Health Scale***

**Observations:**

# PLEASE CHECK THAT YOU HAVE ANSWERED ALL THE QUESTIONS.

# THANK YOU VERY MUCH FOR YOUR TIME AND COOPERATION.
